# Assessing the Effect of the Anti-tuberculosis Drug Rifampicin on Known Hypertensive Patients With Tuberculosis in a Tertiary Care Center

**DOI:** 10.7759/cureus.49701

**Published:** 2023-11-30

**Authors:** Yogesh S, Naveenkumar Nallathambi, Ganapathy Raja K, Hariharan Seshadri, Gautham R, Shriganesh P Naidu, Navvin S, Preetham Ezhilarasu, Ahimth JA, Suriya Prakash Srinivasan

**Affiliations:** 1 Internal Medicine, Madras Medical College and Rajiv Gandhi Government General Hospital, Chennai, IND

**Keywords:** tuberculosis (tb), treatment-resistant hypertension, medication interaction, systemic hypertension, anti-hypertensives, anti-tuberculosis therapy, rifampicin

## Abstract

Background

Epidemiological evidence suggests an indirect link between hypertension and tuberculosis, and several studies have reported that rifampicin has potentially diminished the hypotensive effects of many anti-hypertensive agents by inducing cytochrome P450. This study investigates rifampicin’s effect on the target blood pressure in known hypertensive patients whose blood pressure had been previously controlled with anti-hypertensive drugs.

Methodology

This prospective observational study was conducted at the Institute of Internal Medicine, Madras Medical College and Rajiv Gandhi Government General Hospital, Chennai, from June 2021 to December 2022. A total of 160 patients with known hypertension on anti-hypertensive drugs were recruited for this study. All these patients had been recently diagnosed with tuberculosis and had been treated with rifampicin-based anti-tuberculosis therapy (ATT).

Results

The maximum number of patients were under 50 years of age and predominantly male (67%). A total of 91 (57%) patients were hypertensive for less than five years, and the remaining patients were hypertensive within 6-10 years or more than 10 years. However, these patients had other comorbidities such as diabetes mellitus (32%) and coronary artery disease (27%). Before ATT, the mean systolic blood pressure (SBP)/diastolic blood pressure (DBP) was recorded to be 130/80 mmHg. The last six months’ course of ATT showed mean values around 154/96 mmHg even after adding additional/multiple anti-hypertensive drugs. After discontinuation of ATT, the mean SBP/DBP was effectively 130/80 mmHg at four weeks.

Conclusions

Rifampicin significantly diminishes the hypotensive effects of many well-established anti-hypertensives such as calcium channel blockers, beta-blockers, and diuretics to maintain blood pressure.

## Introduction

Lifestyle factors are well-known to affect the risk of hypertension and cardiovascular disease (CVD). However, chronic inflammatory infections such as tuberculosis may also act as causal factors for the development of hypertension by triggering various immunological responses, leading to endothelial dysfunction and increased risk of hypertension and CVD. The association between arterial hypertension and tuberculosis remains unclear. Rapid urbanization, improving socioeconomic status, decreased morbidity and mortality from infectious disease, and evolving and changing lifestyle and dietary habits have led to a rapid rise in non-communicable diseases, especially hypertension, diabetes mellitus, malignancy, and CVD. Hypertension is a crucial risk factor for cardiovascular-related death, cerebrovascular accidents, and kidney-related disease [1]. A systemic review of many studies found no evidence supporting an association between hypertension and tuberculosis. Aside from a possible direct link between renal tuberculosis and renal failure resulting in hypertension, no other association has been found.

Nevertheless, hypertension may indirectly affect the immune system, which could increase the risk of tuberculosis in patients, which has also been shown to increase the risk of CVD [2]. Epidemiological evidence suggests an association between diabetes and tuberculosis infection, possibly because of immune impairment leading to increased susceptibility to active tuberculosis infection [3,4]. The World Health Organization recommended formulations of anti-tuberculosis therapy (ATT) as fixed-dose combinations to include isoniazid, rifampicin, pyrazinamide, and ethambutol, and the duration of treatment is based on whether it is pulmonary tuberculosis or extrapulmonary tuberculosis ranging from six to eighteen months [5].

Rifampicin is the first-line anti-tuberculosis drug and an antibiotic used to treat many bacterial infections [6]. It is the most influential enzyme inducer of hepatic cytochrome P450 enzymes that lead to increased metabolism of many drugs and turns them into ineffective drugs such as warfarin, birth control pills, antiretrovirals, immune suppressants, as well as some anti-hypertensives such as calcium channel blockers, beta-blockers, and diuretics [7]. Several studies have reported that rifampicin has potentially diminished the hypotensive effects of many anti-hypertensive agents by inducing cytochrome P450 [8-10]. Thus, this study aims to investigate the effect of rifampicin on the target blood pressure in known hypertensives whose blood pressure had been previously controlled with anti-hypertensives.

## Materials and methods

Study design and setting

This prospective observational study was conducted at the Institute of Internal Medicine, Madras Medical College and Rajiv Gandhi Government General Hospital, Chennai, from June 2021 to December 2022. A total of 160 patients with known hypertension on anti-hypertensive drugs were recruited for this study. All these patients had been recently diagnosed with tuberculosis and were treated with rifampicin-based ATT.

Inclusion criteria

Patients above 18 years of age with microbiologically/clinically confirmed tuberculosis on ATT and those previously diagnosed as hypertensives taking dual and triple anti-hypertensive treatment were included.

Exclusion criteria

Patients under 18 years of age and those with a history of liver diseases, renal failure, coarctation of the aorta, pheochromocytoma, renal artery stenosis, Cushingoid, and acromegalic features were excluded.

Ethical considerations

Written approval from the Institutional Ethics Committee of Madras Medical College, Chennai was obtained beforehand (approval number: 13072021). Written approval from the medical department and related departments was obtained. After obtaining informed verbal consent, all cases with a confirmed diagnosis of tuberculosis taking anti-hypertensive drugs were included.

Study methodology

Recruited patients were subjected to clinical and physical examination, and all records were collected. All recruited patients underwent routine investigations to rule out secondary hypertension, including complete blood count, renal function test, liver function test, serum electrolytes, ultrasound of the kidney and urinary bladder, and echocardiogram. Patients’ blood pressure was measured before starting ATT and was considered the baseline blood pressure. A continuous ambulatory blood pressure monitor monitored the patient’s blood pressure (for three days). Both systolic blood pressure (SBP) and diastolic blood pressure (DBP) were measured for all patients at three intervals, namely, pre-ATT with two follow-up baselines within one week; during ATT with four follow-up periods of two weeks, two months, four months, and six months; and post-ATT with two follow-ups at two weeks and four weeks. The multiple mean blood pressure values recorded were necessary for three different aspects, namely, to ensure adequate control of blood pressure by either increasing the doses of existing drugs or adding additional drugs if necessary; to understand the dosage and quantity if the need for antihypertensives diminishes after completion of ATT treatment. During this study period, to maintain blood pressure, patients were given needed additional/multiple anti-hypertensive medications such as calcium channel blockers, angiotensin-converting enzyme inhibitors/angiotensin receptor blockers, beta-blockers, thiazide diuretics, loop diuretics, potassium-sparing diuretics, and alpha 1 blockers at pre-ATT, during ATT, and post-ATT. Additional drugs were added in lieu of increasing blood pressure recordings by the investigator at his discretion.

Statistical analysis

Data collected were entered in Microsoft Excel (Microsoft Corp., Redmond, WA, USA) and analyzed using SPSS version 24.0 (IBM Corp., Armonk, NY, USA) using paired t-test.

## Results

A total of 160 known hypertensive patients were recruited for this study. The majority of patients were <50 years of age were predominantly male (67%). A total of 91 patients were hypertensive for <5 years (57 %), and the remaining patients were hypertensive for 6-10 years or >10 years. However, these patients had other comorbidities such as diabetes mellitus (32%) and coronary artery disease (27%) (Table 1).

**Table 1:** Baseline characteristics of all known hypertensive patients.

Variables		Frequency (n = 160)	Percentage
Age group	<40	60	37.5
	41–50	58	36.3
	51–60	19	11.9
	>61	23	14.4
Gender	Male	108	67.5
	Female	52	32.5
Hypertension duration (in years)	<5	91	56.9
	6–10	58	36.3
	>10	10	6.9
Comorbidities			
Diabetes mellitus	No	109	68.1
	Yes	51	31.9
Coronary artery disease	No	117	73.1
	Yes	43	26.9

Mean systolic and diastolic blood pressure at pre-, during, and post-ATT

The mean SBP measured before starting ATT (baseline) was 130 mmHg. During ATT, the mean SBP for two weeks, two months, four months, and six months was 132 mmHg, 140 mmHg, 148 mmHg, and 154 mmHg, respectively. However, after discontinuation of ATT, mean SBP levels were measured at two weeks and four weeks as 136 mmHg and 130 mmHg, respectively. Regarding DBP measured before ATT, baseline mean values were 80 mmHg. While on ATT, the two-week mean level was 90 mmHg, whereas the mean value at two months was 94 mmHg, 95 mmHg at four months, and 96 mmHg at six months. Post-ATT mean DBP value at two weeks was 84 mmHg, and four weeks later, it was 80 mmHg. Compared to a baseline value of SBP and DBP, there was a progressive rise in blood pressure at six months of ATT in both SBP and DBP, and the mean blood pressure values (both systolic and diastolic) fell after discontinuing ATT, returning close to the baseline at the end of four weeks post-ATT (Table 2). All results showed that at six months of ATT, the mean deviation was 3.18 compared to the baseline which was statistically significant, with the maximum deviation. With the discontinuation of ATT, the mean deviation values returned to the baseline at four weeks.

**Table 2:** Mean deviation of systolic blood pressure and diastolic blood pressure at pre-, during, and post-ATT. ATT: anti-tuberculosis therapy

Blood pressure	ATT	Follow-up	Mean	Standard deviation
Systolic blood pressure	Pre-ATT	Baseline	130.26	6.74
	During ATT	2 weeks	132.19	8.2
		2 months	139.54	7.59
		4 months	148.61	7.95
		6 months	153.42	6.98
	Post-ATT	2 weeks	135.26	6.74
		4 weeks	130.26	6.74
Diastolic blood pressure	Pre-ATT	Baseline	81.26	7.16
	During ATT	2 weeks	90.91	7.22
		2 months	94.08	7.29
		4 months	95.16	7.64
		6 months	96.24	8.21
	Post-ATT	2 weeks	83.62	7.16
		4 weeks	80.26	7.16

Number of anti-hypertensives required for patients at pre-, during, and post-ATT

In this study, recruited patients were prescribed one to three anti-hypertensive drugs (maximum dose) to maintain their blood pressure. To maintain their blood pressure at <130/80 mmHg before starting ATT at baseline, 88 patients took one anti-hypertensive, whereas 54 and 18 patients took two to three different anti-hypertensives (Table 3).

**Table 3:** Number of anti-hypertensive drugs required for patients at pre-, during, and post-ATT. ATT: anti-tuberculosis therapy

ATT	Number of drugs	Frequency	Percentage
Pre-ATT	1	88	55
	2	54	33.8
	3	18	11.3
During ATT			
2 weeks	1	81	50.6
	2	46	28.8
	3	33	20.6
2 months	1	65	40.6
	2	51	31.9
	3	25	15.6
	4	19	11.9
4 months	1	25	15.6
	2	77	48.1
	3	32	20
	4	12	7.5
	5	14	8.8
6 months	1	5	3.1
	2	37	23.1
	3	72	45
	4	26	16.3
	5	11	6.9
	6	9	5.6
Post-ATT			
2 weeks	1	77	48.1
	2	59	36.9
	3	15	9.4
	4	9	5.6
4 weeks	1	92	57.5
	2	59	36.9
	3	9	5.6

For optimal blood pressure control, most (55%) of the patients pre-ATT required only one anti-hypertensive medication, and 33% only needed two anti-hypertensive drugs (Table 4). During ATT, the anti-hypertensive requirements ranged from one to six to maintain blood pressure at <130/80 mmHg. After two weeks of ATT, several patients required one, two, and three different anti-hypertensives 81, 46, and 33, respectively. The requirement for one anti-hypertensive was 65 patients at two months, 25 at four months, and five at six months. Whereas the number of patients requiring two anti-hypertensives was 51 at two months, 77 at four months, and 37 at six months. The number of patients requiring three anti-hypertensives was 25 at two months, 32 at four months, and 72 at six months. During the course of ATT, four anti-hypertensives were required for 19 patients at two months, 12 at four months, and 26 at six months. However, two months of ATT did not require more than four anti-hypertensives, whereas 14 patients at four months and 11 patients at six months required five different anti-hypertensives. At four months of ATT, patients did not require more than five anti-hypertensives. During six months of ATT, nine patients still required about six different anti-hypertensives to maintain their blood pressure. At the end of six months of ATT, 46 patients had required more than three anti-hypertensives (Table 3). During ATT, about 45% of the patients in the study population needed more than four anti-hypertensive drugs to attain optimal blood pressure (Table 4).

**Table 4:** Distribution of anti-hypertensive drugs required for patients at pre-, during, and post-ATT. ATT: anti-tuberculosis therapy

Number of anti-hypertensives required	1	2	3	4	>5
Pre-ATT	55%	33%	12%	-	-
During ATT					
2 weeks	50%	28%	22%	-	-
2 months	40%	32%	16%	12%	-
4 months	16%	48%	20%	8%	8%
6 months	3%	23%	45%	16%	13%
Post-ATT					
2 weeks	48%	37%	10%	5%	-
4 weeks	56%	37%	7%		

After completion of ATT (post-ATT), at two weeks, the number of patients who required one, two, three, and four anti-hypertensives was 77, 59, 15, and 9, respectively. However, at four weeks, the number of patients requiring one anti-hypertensive increased to 93, whereas two anti-hypertensives were needed by 59 patients, and three other antihypertensives were needed by nine patients (Table 3). Post-ATT (after one month) showed a majority (57%) of the patients requiring only one anti-hypertensive drug for optimal blood pressure control (Table 4).

## Discussion

Rifampicin is a potent enzyme inducer of both intestinal and hepatic cytochrome p450. It can diminish the hypotensive effects of many well-established anti-hypertensives such as calcium channel blockers, beta-blockers, and diuretics via the hepatic cytochrome P450 pathways. Hence, monitoring high blood pressure is recommended for patients receiving rifampicin-based ATT to prevent hypertension-related complications such as CVD, cerebrovascular accidents, and renal failure. Therefore, in this study, potential drug-drug interactions between ATT (rifampicin) and antihypertensive drugs (calcium channel blockers, beta-blockers, and diuretics), especially seen by the temporal relationship between the six-month course of ATT and the occurrence of more resistant hypertension cases requiring additional/multiple antihypertensive medications, were investigated.

Similar to our result, Agrawal et al. conducted a prospective study with 24 hypertensive chronic kidney disease (CKD) patients newly diagnosed with tuberculosis, followed by rifampicin-based ATT. Blood pressure was initially controlled (<140/90 mmHg) with one or two different classes of anti-hypertensives. Serum levels of amlodipine, metoprolol, and prazosin were measured on days three, seven, 10, and 14 after the initiation of rifampicin. All 24 patients in the prospective study had worsening hypertension, and 85% required more drugs to maintain blood pressure at <130/80 mmHg, and serum levels of amlodipine, metoprolol, and prazosin declined by >50% in a majority of the patients. The requirement of the anti-hypertensives also increased from 4.6 ± 3.5 units to 8.6 ± 6.5, which was statistically significant (p < 0.001). Of the 24 patients, four developed pulmonary edema, whereas three discontinued rifampicin to achieve blood pressure control. Thus, the study concluded that CKD patients who are hypertensive and were started on ATT need stringent blood pressure monitoring [11].

In our study, although we were unable to measure the drug levels, the results were in concordance with their research in the form of patients needing more anti-hypertensive drugs to control blood pressure during ATT and difficulty attaining optimal blood pressure control. After the withdrawal of rifampicin, blood pressure fell in all patients, and the doses of the anti-hypertensive agents had to be reduced. These findings indicate that rifampicin may reduce the anti-hypertensive effects of dihydropyridine calcium channel blockers [11,12].

These results concord with our study in the form of patients needing much higher doses of calcium channel blockers or additional antihypertensive drugs to control blood pressure during ATT and difficulty attaining optimal blood pressure control [11-14]. All these results showed the potential interaction of rifampicin with the anti-hypertensive drugs of different classes for attaining optimal blood pressure control in our study population.

## Conclusions

Clinicians should be concerned about the possible interactions between rifampicin and other well-known anti-hypertensive drugs. Antibiotics such as rifampicin, which are frequently used to treat bacterial infections and tuberculosis, have been shown to reduce the effects of a number of anti-hypertensive medication types, such as beta-blockers, calcium channel blockers, and diuretics. The primary mechanism underlying this interaction is the activation of hepatic enzymes by rifampicin, specifically cytochrome P450, which can expedite the metabolism and excretion of these anti-hypertensive medications from the body. Interactions impair blood pressure regulation, which raises the possibility of uncontrolled hypertension and the cardiovascular problems that go along with it. To maintain the best possible blood pressure control, clinicians should exercise caution when administering rifampicin to patients who also have concomitant hypertension. Ultimately, this knowledge can improve patient outcomes by promoting safer and more efficient pharmacotherapy.

## Appendices

Interview questions

Patient details

**Table 5:** Patient details.

Particulars	Details
Name:	
Age:	
Sex:	

Clinical history

Tuberculosis history

**Table 6:** Tuberculosis history.

History	Response
Fever duration	
Loss of weight/Loss of appetite	Yes/No
Swelling in the neck	
Respiratory - cough/shortness of breath/chest pain/hemoptysis	
Neurological - altered sensorium/seizures	
Abdominal pain	
H/o months of taking anti-tuberculosis therapy	

Hypertension history

**Table 7:** Hypertension history.

History	Response
Duration	
Anti-hypertensives with dosage:	
Baseline blood pressure:	
Compliance with drug intake:	Yes/No

General examination

**Table 8:** General examination.

Parameters	Findings
Consciousness, orientation to time, place, and person	
Pallor	
Icterus	
Cyanosis	
Clubbing	
Lymphadenopathy	
Pedal edema	

Vitals

**Table 9:** Vitals.

Parameters	Values
Blood pressure (mmHg)	
Pulse rate (per minute)	
Respiratory rate (per minute)	
Temperature (°C)	
SpO_2_ (in room air)	

Clinical examination

**Table 10:** Clinical examination.

Systems	Significant findings
Cardiovascular system:	
Respiratory system:	
Abdomen:	
Central nervous system:	

Investigations

**Table 11:** Investigations.

Parameters	Findings
Complete hemogram	
Renal function tests	
Liver function tests	
Electrolytes	
Chest X-ray/ CT chest	
Echocardiography	
USG abdomen	
Gene-XPERT	

Anti-hypertensives and blood pressure before and after taking anti-tuberculosis therapy

**Table 12:** Anti-hypertensives and blood pressure (BP) before and after taking anti-tuberculosis therapy (ATT).

Antihypertensive drug	Baseline BP before taking ATT	BP after 2 weeks of ATT	BP after 2 months of ATT	BP after 4 months of ATT	BP after 6 months of ATT	BP after 2 weeks of stopping ATT	BP after 4 weeks of stopping ATT
Drug 1							
Drug 2							
Drug 3							
Drug 4							
Drug 5							
